# Fruit Quality Evaluation Using Spectroscopy Technology: A Review

**DOI:** 10.3390/s150511889

**Published:** 2015-05-21

**Authors:** Hailong Wang, Jiyu Peng, Chuanqi Xie, Yidan Bao, Yong He

**Affiliations:** College of Biosystems Engineering and Food Science, Zhejiang University, Hangzhou 310058, China; E-Mails: hl_wang@zju.edu.cn (H.W.); jypeng@zju.edu.cn (J.P.); cqxie@zju.edu.cn (C.X.)

**Keywords:** Vis/NIR, fruit quality, chemometrics, discrimination, characteristic wavelength, SSC, acidity

## Abstract

An overview is presented with regard to applications of visible and near infrared (Vis/NIR) spectroscopy, multispectral imaging and hyperspectral imaging techniques for quality attributes measurement and variety discrimination of various fruit species, *i.e.*, apple, orange, kiwifruit, peach, grape, strawberry, grape, jujube, banana, mango and others. Some commonly utilized chemometrics including pretreatment methods, variable selection methods, discriminant methods and calibration methods are briefly introduced. The comprehensive review of applications, which concentrates primarily on Vis/NIR spectroscopy, are arranged according to fruit species. Most of the applications are focused on variety discrimination or the measurement of soluble solids content (SSC), acidity and firmness, but also some measurements involving dry matter, vitamin C, polyphenols and pigments have been reported. The feasibility of different spectral modes, *i.e.*, reflectance, interactance and transmittance, are discussed. Optimal variable selection methods and calibration methods for measuring different attributes of different fruit species are addressed. Special attention is paid to sample preparation and the influence of the environment. Areas where further investigation is needed and problems concerning model robustness and model transfer are identified.

## 1. Introduction

Over the last couple of decades, with the rapid development of the economy and improvement of living standards, fruit consumption has increased significantly. Meanwhile, consumers have higher expectations of fruit qualities such as ripeness, firmness, soluble solids content (SSC) and acidity. However, many fruit quality attributes affecting consumer acceptance and price are still tested using traditional approaches which are either subjective or time-consuming, so it should be a surprise that how to measure fruits’ internal and external attributes nondestructively and rapidly has become a research hotspot. Researchers all over the world have investigated the potential of various technologies, including acoustic techniques, spectroscopic techniques, machine vision and electronic noses, for the assessment of fruit qualities. Among all these technologies, spectroscopic techniques have drawn great attention for their prominent advantages: (1) they are nondestructive methods which enable the acquisition of fruits’ internal quality parameters without damaging their surfaces; (2) the measurement processes are simple and rapid, as no complex pretreatments or chemical reactions on fruit samples are needed; (3) they enable the detection of several fruit internal attributes simultaneously. As a disadvantage, however, the small point-source measurements which are commonly used in spectral assessment cannot provide spatial information, which is important in many fruit quality evaluation instances.

Imaging and spectroscopy are two important directions of conventional optical technology. Imaging techniques can obtain the images of fruits and acquire their spatial information while spectroscopy provides access to information about the chemical components and physical properties of fruits by obtaining optical information. Imaging spectral techniques enable the acquisition of fruit images and spectral information simultaneously, with the advantages of high spectral resolution and multiple wavebands. According to the spectral resolution, imaging spectroscopy can be divided into multispectral imaging, hyperspectral imaging and ultra-spectral imaging. Multispectral imaging and hyperspectral imaging are proved to be feasible for the measurement of fruit quality parameters. However, few papers concerning both imaging technique and spectroscopy technique can be found yet, so in this review, most attention was paid to spectroscopic techniques, rather than imaging techniques.

Visible and near infrared (Vis/NIR) radiation covers the range from 380–2500 nm in the electromagnetic spectrum. As the signals of almost all major structures and functional groups of organic compounds can be detected in the Vis/NIR spectrum with a considerably stable spectrogram, spectra in the Vis/NIR range are frequently used for analysis [1]. Wavebands which are commonly used in multispectral and hyperspectral imaging technologies to assess fruit quality are also in the Vis/NIR region [2,3,4,5]. When incident radiation hits a sample, it may be reflected, transmitted or absorbed. Correspondingly, a spectrum is obtained in the reflectance, transmittance or absorbance mode, each of which can reflect some physical attribute and chemical constitution of the sample.

After the spectrum is obtained, chemometric methods are applied to extract information concerning the quality attributes and to eliminate the interference of factors irrelevant to sample concentration. In general, chemometrics consist of two parts, spectral pretreatments and regression methods.

The objective of this review is to offer a comprehensive overview of the use of Vis/NIR spectroscopy, multispectral imaging and hyperspectral imaging techniques in the measurement of various fruit quality attributes. We will briefly introduce the chemometric methods commonly used, and pay extra attention to the identification of optimal methods for variable selection and quality measurement.

## 2. Chemometrics

Applications of spectroscopy as well as multispectral and hyperspectral imaging technologies to measure fruit quality attributes are usually carried out in the Vis/NIR region since spectra in this range incorporate abundant information concerning O-H, C-H and N-H vibration absorptions [6]. However, in this region, the spectrum is basically dominated by water which highly absorbs near infrared radiation [7]. Besides, the Vis/NIR spectrum has a low signal-to-noise ratio and high overlap of combination bands and overtones, not to mention the complex constitution of fruits, wavelength-dependent light scattering and instrumental noise. All these cause the convolution of the Vis/NIR spectrum. Therefore, chemometrics are applied for extracting information concerning certain quality attributes from the spectral data.

### 2.1. Pretreatment Methods

#### 2.1.1. Smoothing

Smoothing is an effective approach for removing high-frequency noise from a spectrum and improving the signal-to-noise ratio. Its basic idea is to obtain an optimal estimation value through the “averaging” or “fitting” of several points in a window. The broader the window is, the lower the spectral resolution would be. Thus it is crucial to choose the window width properly. Based on different smoothing fit methods, smoothing could be divided into moving average smoothing, Gaussian filter smoothing, median filter smoothing and Savitzky-Golay smoothing (S-G smoothing). Sun *et al.* [8] proved that moving average smoothing was the most feasible pretreatment method for SSC prediction of navel oranges, and Roger and Bellon-Maurel [9] applied NIR spectra processed with moving average smoothing for the measurement of sugar content in cherry fruit. However, smoothing is usually used in combination with other pretreatment methods, such as Multiplicative Scatter Correction (MSC). Liu and Zhou [10] claimed that the combination of 1st derivative, MSC and smoothing was feasible to process Vis/NIR transmittance spectra for predicting SSC in apples.

#### 2.1.2. Offset Correction

This is a centralized processing method which is realized by subtracting the average value of the first few wavelength points (for example five) from each spectrum. Offset correction only adjusts the baseline drift, leaving the spectrum shape unchanged. It is mainly used for weakening the influence of instrumental noise, optical distance and detection environment.

#### 2.1.3. De-Trending

De-trending is an approach to eliminate the baseline drift in the spectrum. Firstly a trend line was derived from spectral values and wavelengths through least squares fitting, and then the trend line was subtracted from the original spectrum. De-trending is often used in combination with standard normal variate correction (SNV), which we could find in the studies of Sanchez *et al.* [11] who predicted firmness in strawberries and Paz *et al.* [12], who predicted SSC and firmness in plums.

#### 2.1.4. Multiplicative Scatter Correction (MSC)

First proposed by Ilari *et al.* [13], MSC was used to compensate the effect of non-uniform scattering induced by diverse particle sizes, uneven distribution and other physical effects in the spectral data. MSC is performed by linearizing each spectrum to an “ideal” spectrum, which corresponds to the average spectrum of the calibration set. The linear relationship between each spectrum and the average spectrum is fitted through the method of least squares. This suggests that MSC is feasible for removing the ‘ideal’ linear scattering and effects well when the linear relationship between absorbance and sample concentration is good. The feasibility of MSC was already confirmed by Liu *et al.* [14] and Shao *et al.* [15].

#### 2.1.5. Standard Normal Variate (SNV)

Basically the same as MSC, the objective of SNV is to eliminate the deviations caused by particle size and scattering [16]. The method assumes that the absorbance of each wavelength point in the spectrum meets some certain distribution such as a Gaussian distribution. Based on this hypothesis, each spectrum is calibrated. Firstly the average value of a spectrum is subtracted from the original spectrum, and then the result is divided by the standard deviation (*SD*). For SNV effects on each spectrum alone, the correction capability of SNV is usually stronger than that of MSC. In the model established by Shi *et al.* [17] to evaluate the firmness of apples, the relative standard deviation of prediction (*RSDP*) was reduced from 16.65% to 14.82% after SNV processing.

#### 2.1.6. Derivative Correction

As a widely-used pretreatment method, first and second derivatives are applied to eliminate drifting and scattering, respectively. They can remove background interference, distinguish superposed peaks and enhance the spectral resolution and sensitivity. Two commonly-used spectral derivative approaches are direct finite difference and Savitzky-Golay (S-G) derivatives. Before derivatization, smoothing should be applied because derivatives may extract differences of adjacent wavelength points and amplify spectral noise. Pissard *et al.* [6] proved S-G 1st derivative processing was the best pretreatment method, while Liu *et al.* [18] claimed that the 2nd derivative was the best.

#### 2.1.7. Wavelet Transformation (WT)

Introduced and applied in the study of Liu *et al.* [19], WT is an emerging signal and image processing method. In spectral analysis, WT is often used for data compression, smoothing and filtering, as well as the extraction of effective information. By applying a basis function, chemical signals can be decomposed into various scale compositions according to their different frequencies. Sampling windows of corresponding width are applied to scale compositions of different sizes, thus any part of the signal could be focused on. Narrow windows could be used to observe drastic changes while wide windows could be used to observe the overall features of the spectrum. Among the various wavelet functions, some were proved to be quite effective, such as Daubechies and Symlets. Xia *et al.* [20,21] chose Daubechies 3 while Shao and He [22] applied Daubechies 2.

#### 2.1.8. Orthogonal Signal Correction (OSC)

When there’s little correlation between the spectral matrix and the concentration matrix of certain quality attributes or the background noise is big, the first several principal components (PCs) selected by PLS or PCA contain very limited information about the concentration matrix. Eliminating these irrelevant signals before calibration through some orthogonal approaches could effectively reduce the number of PCs and enhance the prediction ability as well as stability of the calibration model. Therefore, OSC was introduced to calibration transfer by Sjoblom *et al.* [23]. In addition, OSC could also be applied to solve problems concerning model transfer and outlier detection. Its outstanding ability of improving the prediction ability was proved by Shi *et al.* [17], who applied direct OSC (DOSC) in his study.

#### 2.1.9. Net Analyte Preprocessing (NAP)

Introduced by Goicoechea *et al.* [24], NAP is mainly used for extracting spectral information concerning a certain ingredient in the spectra of the mixture. The feasibility of NAP was proved by Lv *et al.* [25].

### 2.2. Variable Selection Methods

Due to the abundance of information and severe nonlinearity in the full spectrum, some processing methods have been applied on the original spectral data in the whole wavelength range to extract characteristic wavelengths (CWs) with the highest predictive ability. In this way the number of input variables is reduced and the calibration time is shortened.

#### 2.2.1. Successive Projections Algorithm (SPA)

Introduced by Araujo *et al.* [26], SPA is a forward selection method searching for a group of variables with minimum redundant information and minimal collinearity through simple operations in a vector space. Starting with one wavelength, SPA incorporates a new wavelength during each iteration until a certain number is reached. The CWs extracted by SPA could represent the spectral information of most samples and avoid information overlap at the highest degree possible. In the study of Zhang *et al.* [27], the Least Squares Support Vector Machine (LS-SVM) model combined with SPA could yield a result better than that of the LS-SVM for full spectrum.

#### 2.2.2. Regression Coefficient (RC)

RCs could be obtained during the calibration of partial least squares regression (PLS) [28]. The RC value corresponding to each wavelength point represents its ability to affect the predictive performance of the model. Thus based on the absolute value of RCs, CWs could be identified.

#### 2.2.3. Loading Weights (LW)

Loading weights could also be obtained during the calibration of PLS. Under each latent variable, loading weights corresponding to wavelengths could be obtained and their absolute values illustrate the wavelengths’ impact on the prediction model, so a wavelength with the maximum loading weight value is selected as the CW and the number of CWs is the same of the number of latent variables. Fernandez-Novales *et al.* [29] used CWs selected by the loading weights of latent variables to build a MLR model for sugar content prediction and obtained a satisfactory result.

#### 2.2.4. Genetic Algorithm (GA)

As an effective global searching method, GA mimics the competitive mechanism of survival of the fittest in biological world. Based on a fitness function, GA is an iterative process starting from a population of randomly generated individuals and achieves optimal solutions through genetic operations including crossover, selection and mutation. When GA is applied for variable selection, the number of iterations is set, usually above 100 and root mean square error for cross validation (*RMSECV*) is often used as the fitness function. After the iteration, the variables are realigned based on the frequency they are selected. The variable with the highest frequency is used for calibration and one more variable is included each time sequentially. The optimal number of variables used is determined when a minimized *RMSECV* is achieved. Through such operation, irrelevant spectral information is eliminated and the number of spectral variables is reduced. Cao *et al.* [30] executed GA for the selection of CWs for predicting SSC in grapes and yielded a satisfactory result.

#### 2.2.5. Competitive Adaptive Reweighted Sampling (CARS)

CARS is a novel wavelength selection method introduced by Li *et al.* [31]. Wavelengths with large absolute coefficients are sequentially identified as the CWs based on the adaptive reweighted sampling technique in a PLS model. A series of variable subsets are obtained and cross validation is employed to choose the optimal one with the lowest *RMSECV*. Sun *et al.* [8] validated that the CWs selected with CARS could yield the best result.

#### 2.2.6. Uninformative Variables Elimination (UVE)

UVE was first proposed by Centner *et al.* [32] and its basic approach is to add some noise variables into the experimental variables and calibration models are built with the mixed variables. The importance of each variable is evaluated and the experimental variables with no more importance than the noise variables are eliminated. The UVE method was employed by Sun *et al.* [8].

Readers are referred to the corresponding references for details about many other effective variable selection methods that we do not introduce in detail here, including backward interval PLS (BiPLS) [33], synergy interval PLS (siPLS) [34,35], independent component analysis (ICA) [36], simulated annealing algorithm (SAA) [37] and stepwise regression analysis (SRA) [27].

### 2.3. Discriminant Methods

#### 2.3.1. Principal Component Analysis (PCA)

With the application of PCA, a set of principal components (PCs) are obtained. The first PC contains the largest percentage of data variance and the variance decreases in the following PCs. These PCs are linear combinations of the original spectral data but uncorrelated with each other, endowing their ability to handle multicollinearity. PCA is often utilized in combination with other discriminant methods [38,39,40,41].

#### 2.3.2. Partial Least Squares-Discriminant Analysis (PLS-DA)

Introduced by Liu *et al.* [42], PLS-DA is a method commonly applied for optimal classification. Based on PLS regression, PLS-DA uses dummy variables such as 1, 2, 3, *i.e.*, as variables of Y matrix instead of the concentration of some quality attributes. The optimal number of PLS components are decided by full cross-validation. The feasibility of PLS-DA was proved by Cen *et al.* [39] and Hao *et al.* [43].

#### 2.3.3. Soft Independent Modeling of Class Analogy (SIMCA)

When SIMCA is applied, a PCA model is established for each class in a certain training data set. Then each observation is assigned to a class based on its residual distance from the model. However, each model is established independently without consideration of other classes. Due to the overlapping between classes, there’s a chance of producing a non-optimized discriminant model. SIMCA was employed by Cao *et al.* [40], Baranowski *et al.* [44] and Hao *et al.* [43].

#### 2.3.4. Linear Discriminant Analysis (LDA)

Introduced and used by Baranowski *et al.* [44], LDA is commonly used in machine learning to search for a linear combination of characteristics separating different classes of objects. It offers a linear transformation of n-dimensional feature vectors into an m-dimensional space (m < n). This linear combination could be used as a classifier or for data dimensionality reduction.

#### 2.3.5. Support Vector Machine (SVM)

Like SIMCA and LDA, SVM is a pattern recognition method which is quite useful for supervised classification. It is feasible to both linear and nonlinear data, by using kernel function, which maps from the original space to the feature space and guarantees the ability to handle nonlinear classification. With the use of statistical learning, a hyperplane for optimal discrimination is determined. The feasibility of SVM has been proved by Baranowski *et al.* [44] and Guo *et al.* [45]. Other commonly applied discriminant methods include stepwise discriminant analysis (SDA) [46,47], and BP-ANN [15,48,49,50].

### 2.4. Calibration Methods

#### 2.4.1. Multiple Linear Regressions (MLR)

MLR predicts the dependent variables by a linear combination of spectral values at each wavelength point. The error between predicted and measured values is minimized in a least squares sense. In spectral analysis, multicollinearity between the variables degrades the performance of MLR algorithms. MLR was successfully employed by Peiris *et al.* [51] and ElMasry *et al.* [52]. However, Jaiswal *et al.* [53] reported a big gap between *r_c_* and *r_p_* in the MLR model they built, indicating unstable prediction.

#### 2.4.2. Principal Component Regression (PCR)

In PCR, a small number of principal components (PCs) are selected by a principal component analysis (PCA). These PCs are applied as predictors instead of the original spectral data and used to fit a MLR model. PCR was used for calibration in the studies of Park *et al.* [54] and Angra *et al.* [55], with the advantage of eliminating the multicollinearity for the PCs are uncorrelated. However, Hadi and Ling [56] pointed out the potential drawback that the PCs are decided only according to the variables and they may contain little information about the dependent variables.

#### 2.4.3. Partial Least Squares Regression (PLS)

To overcome the drawback of PCR, PLS regression was introduced by Wold *et al.* [57]. It predicts the dependent variables by extracting a smallest possible set of orthogonal factors with greatest predictive abilities from the variables. These orthogonal factors, called latent variables (LVs) were arranged according to the relevance for predicting the dependent variables. Synthesizing the sense of principal component analysis (PCA) and multiple linear regressions (MLR), PLS regression is especially feasible in circumstances where multicollinearity exists between the variables and the number of latent variables is usually smaller than that in the PCR regression. The advantage toward PCA was confirmed by Liu *et al.* [58] and Lu *et al.* [59]. Lots of researchers have applied PLS in their studies, including Shan *et al.* [5] and Bureau *et al.* [60].

#### 2.4.4. Least Squares Support Vector Machine (LS-SVM)

LS-SVM is an emerging statistic learning algorithm which improves the generalization ability of the learning machine based on the principle of structural risk minimization [42]. The computational complexity and quality of the support vector machine does not directly depend on the dimension of input data. Therefore, LS-SVM is widely applied in pattern recognition and function regression for the advantage of limited over-fitting, high predictive reliability and strong generalization ability. LS-SVM is especially feasible for circumstances of small sample space modeling. LS-SVM was applied as the best calibration method in the studies of Suykens and Vanderwalle [61], Zhang *et al.* [27], Liu and Zhou, Pissard *et al.* [10] and Liu *et al.* [62].

#### 2.4.5. Artificial Neural Network (ANN)

ANN has been widely used in NIR calibration. Usually an ANN model consists of three layers of neurons, which are the input layer, the hidden layer and the output layer. Each neuron in the previous layer is connected to each neuron in the latter layer and every connecting line has a weight factor, the value of which is assessed based on a calibration set using cross validation and keeps changing with the influx of new information. The value of neurons in the hidden layer is decided by weighted sum of values of neurons in the input layer using a nonlinear function and the value of neurons in the output layer is decided by the values of neurons in the hidden layer similarly. In certain circumstances the predictive performance of the ANN model may be excellent, but it also faces drawbacks such as slow training speed, over-fitting and the visualization difficulty. ANN was proved effective in the studies of Liu *et al.* [63], Zhang *et al.* [64] and He *et al.* [65].

These mentioned above are the most commonly applied calibration methods. Other improved approaches such as Spline-PLS [18] and stepwise MLR [66] have been introduced and utilized by some researchers.

### 2.5. Model Evaluation

The prediction ability of a calibration model is mainly evaluated by the correlation coefficient (*r*) and root mean square error (*RMSEP*) between the predicted value and the measured value in validation set. The higher is the correlation coefficient and the lower is the *RMSEP*, the better is the prediction performance. When cross validation is employed, the prediction performance could also be assessed by the root mean square error for cross validation (*RMSECV*).

Other commonly used evaluation parameters include the standard error of prediction (*SEP*), the standard error of cross validation (*SECV*), the residual predictive deviation (*RPD*) and relative standard deviation (*RSD*). *RPD* is the ratio of standard deviation of the dependent variable to *RMSEP* or *RMSECV*. According to Nicolai *et al.* [7] and Pissard *et al.* [6], for a prediction model, when the *RPD* value is between 2 and 2.5 coarse prediction is possible, while an *RPD* value above 2.5 indicates good to excellent prediction. A similar standard was defined by Davey *et al.* [67], who proved that total carotenoids and β-carotene in banana could be measured accurately (*RPD* = 3.34, 2.74, respectively), α-carotene and c-carotene could be predicted coarsely (*RPD* = 1.68 and 1.96 respectively), and lutein could not be predicted (*RPD* = 1.16).

## 3. Quality Evaluation for Different Fruit Varieties

Spectra in the Vis/NIR range contain abundant information concerning O-H, C-H and N-H vibration absorptions [6], making the measurement of various quality attributes of fruits possible. Some wavebands contain typical absorption bands for some chemical groups. A brief overview was presented in Table 1 to give some guidance for waveband selection.

**Table 1:** Overview of wavebands containing typical absorption bands for certain chemical groups.

Quality Attribute	Chemical Group	Wavelength/nm	Ref.
Sugar	O-H	1190, 1400	[68]
SSC	C-H	910	[69]
	O-H	960, 1450	[14]
	C-H and O-H	1210	
	O-H	975	[70]
	O-H	960, 1180, 1450, 2000	[27]
	C-H	963	
	Combination bands of C-H and O-H	2000–2500	
	O-H and C-H	950–1075	[71]
Acidity	C-O from COOH	1607	[72]
	O-H from carboxyl acids	1127	
	C=O from saturated and unsaturated carboxyl acid	1437	
pH	C-H	768	[73]
	O-H	986	

### 3.1. Apples

Apples are among the most widely cultivated and eaten fruits all over the world. Important attributes affecting the taste of apples include firmness, sugar content and acidity. During the harvest and transport of apples, bruising is inevitable and could affect the quality attributes including appearance, water loss and enhance the risk of bacterial and fungal contamination. The discrimination of bruised apples from the intact ones could ensure the postharvest quality. Other important attributes such as vitamin and polyphenol content also have drawn some attention. A brief overview is presented in Table 2.

**Table 2:** Overview of the applications for measuring quality attributes in apple.

Quality Attribute	Cultivar	Method	Spectral Mode	Spectral Range/nm	Calibration Method	*r_p_*	*SEP*	Ref.
SSC or sugar content	GA	Spectroscopy	Reflectance	800–1100	PCR	0.97	0.28	[54]
	RD			800–1100		0.98	0.34	
	GD	Hyperspectral imaging	Reflectance	500–1000	PLS	0.88	0.7	[4]
	JG					0.78	0.7	
	RD					0.66	0.9	
	Various	Spectroscopy	Reflectance	800–1600	PCR	Unknown	0.73–1.78	[55]
	FJ	Spectroscopy	Transmittance	505–1031	LS-SVM	0.98	0.29	[10]
	Unknown	Spectroscopy	Reflectance	482–1009	PLS	0.96	0.23 (*RMSEP*)	[33]
	FJ	Spectroscopy	Reflectance	833–2500	PLS	0.98	0.69–0.72 (*RMSEP*)	[68]
	FJ	Hyperspectral imaging	Reflectance	480–1016	PLS	0.92	0.67	[5]
	Various	Spectroscopy	Reflectance	400–2500	LS-SVM	0.97	0.37	[6]
	Various	Spectroscopy	Reflectance	5882–9900	PLS	≥0.98	1.9%–3.4% (*RMSEP*)	[60]
Titratable acidity	Various	Spectroscopy	Reflectance	5882–9900	PLS	0.98	6.0% (*RMSEP*)	[60]
Malic acid						0.98	4.7% (*RMSEP*)	
Citric acid						0.34	100% (*RMSEP*)	
Firmness	GA	Spectroscopy	Reflectance	800–1100	PCR	0.47	4.9	[54]
	RD			400–1800		0.89	7.0	
	GD	Hyperspectral imaging	Reflectance	500–1000	PLS	0.87	5.9	[4]
	JG					0.95	7.1	
	RD					0.84	8.7	
	FJ	Spectroscopy	Reflectance	1000–2339	PLS	0.82	14.1 (*RSD*)	[17]
Total polyphenol	Various	Spectroscopy	Reflectance	400–2500	LS-SVM	0.97	140	[6]
	Various	Spectroscopy	Reflectance	6378–9900	PLS	0.98	87.1 (*RMSEP*)	[60]
Vitamin C	Various	Spectroscopy	Reflectance	400–2500	LS-SVM	0.90	0.049	[6]

GA: Gala; RD: Red Delicious; GD: Golden Delicious; JG: Jonagold; FJ: Fuji; IA: Indian apples.

#### 3.1.1. Soluble Solids Content (SSC) or Sugar Content

Park *et al.* [54] found that by using spectra ranging from 800 to 1100 nm, SSC in GA and RD could both be predicted with excellent accuracies (*r_p_* = 0.97, 0.96; *SEP* = 0.28, 0.34, respectively). Angra *et al.* [55] evaluated Brix values of Indian apple along with apples from other countries. Reflectance spectra were acquired at ten wavelengths in the range of 800–1600 nm and the change in spectral reflectance caused by apple shape was eliminated by normalizing the spectral reflectance against a non-absorbing wavelength. SSC values of all cultivars could be predicted with *SEP*s of 0.73–1.78. Liu and Zhou [10] built models with PLS and LS-SVM, respectively, using Vis/NIR transmittance spectra, and the LS-SVM model (*r* = 0.98, *SEP* = 0.29) outperformed the PLS one. The superiority of LS-SVM was proved by Pissard *et al.* [6], who predicted sugar content in more than 150 apple genotypes with Vis/NIR reflectance spectroscopy in the wavelength range of 400–2500 nm. S-G first derivative was proved to be the best data pretreatment and the LS-SVM yielded excellent result, with *r_p_* of 0.97, *SEP* of 0.37°Brix and *RPD* of 4.3. Ouyang *et al.* [33] compared the performance of PLS combined with the backward interval partial least squares method (BiPLS-PLS), genetic algorithm (GA-PLS) and successive projection algorithm (SPA-PLS), respectively. GA-PLS performed the best, with *r_p_* increased from 0.93 to 0.96 and *RMSEP* decreased from 0.30°Brix to 0.23°Brix compared to the PLS model built with full spectrum (482–1009 nm).

Wang *et al.* [74] found the fluctuation of temperature could influence the prediction accuracy in a nonlinear way. When no precautions were taken, the *SEP* of the SSC prediction model could reach as high as 2.55. They offered two methods to enhance the accuracy: a temperature variable-eliminating calibration model and a global robust calibration model, both of which performed well, with *RMSEP* of 0.72 and 0.69, respectively. Bureau *et al.* [60] monitored the change in sugar content during sample preparation. Results showed that different conditions of sample preparation could not affect the sugar concentration. Mid-infrared spectra in the range of 5882–9900 nm showed an excellent ability for predicting sugar content (*r_p_* ≥ 0.98 and *RMSEP* ≤ 3.4%).

Mendoza *et al.* [4] combined spectroscopy with image analysis, reducing the *SEP*s by 11.2, 2.8 and 3.0% (*r_p_* = 0.88, 0.78, 0.66; *SEP* = 0.7%, 0.7%, 0.9%) for GD, JG and RD, respectively. Shan *et al.* [5] observed a slightly better result with hyperspectral imaging in the range of 480–1016 nm for ‘Fuji’ apple. The PLS model based on spectra processed with MSC, 1st derivative and S-G smoothing sequentially yielded an *r_p_* of 0.92 (*SEP* = 0.67°Brix). Zou *et al.* [75] combined a near-infrared spectrophotometer, a machine vision system, and an electronic nose system through ANN to classify ‘Fuji’ apples based on sugar content, making the classification error drop from around 17% when only NIR spectra were used to around 6%.

According to all these studies above, Spectroscopy combined with other measurements performs well in prediction of SSC in apples. However, they all have some drawbacks. NIRS could not obtain the spatial information of samples. The hyperspectral imaging technique had a relatively poor performance in SSC prediction compared with spectroscopy, and the presence of bruises seriously influences the prediction of SSC. MIR shows a good ability to estimate sugar content. However, MIR needs the crushing to sample preparation, which makes it comparatively time consuming. In all, SSC or sugar content of apple could be predicted with good performance though subtle differences existed among cultivars.

#### 3.1.2. Acidity

Bureau *et al.* [60] employed mid-IR spectroscopy in the 5882–9900 nm range to measure organic acids. Through monitoring its quantitative changes, the concentration of organic acids was proved to be unaffected by different conditions of sample preparation, such as storage temperature, sample grinding and sample oxidation. Mid-infrared spectra were feasible for predicting organic acid contents (*r_p_* ≥ 0.98; *RMSEP* ≤ 4.7%), except for citric acid (*r_p_* = 0.75), probably due to its very low content in apple fruit.

#### 3.1.3. Firmness

As shown in Table 2, Park *et al.* [54] predicted the firmness using Vis/NIR diffuse reflectance spectra. According to the PCR models they built, a pretty good result could be obtained for RD using the full spectrum (*r_p_* = 0.89, *SEP* = 7.0). However, for GA, the *r_p_* and *SEP* could only reach 0.47 and 4.9, respectively. In the study of Shi *et al.* [17], after DOSC combined with first derivative was employed to filter out the background and extract useful information, the model was simplified and an acceptable result was obtained (*r_p_* = 0.82; *RSDP* = 14.08%). Mendoza *et al.* [4] employed critical spectral and image features extracted from hyperspectral scattering images in the wavelength range of 500–1000 nm to predict the firmness. Spectral scattering in combination with image features could significantly improve the prediction. The standard error of prediction (*SEP*) for GD, JG and RD apple was reduced by 6.6%, 16.1% and 13.7%, respectively (*r_p_* = 0.87, 0.95, 0.84; *SEP* = 5.9%, 7.1%, 8.7%). These investigations indicated fluctuations in firmness prediction of apple fruit with regard to different varieties. By contrast, hyperspectral imaging shows better performance than spectroscopy in the firmness prediction, though less performance in the SSC prediction.

#### 3.1.4. Total Polyphenols

Pissard *et al.* [6] established an LS-SVM model for the prediction of total polyphenol content in apples using spectra recorded in the 400–2500 nm region. S-G 1st derivative was proved to be the best pretreatment method. The model performed well with *r_p_* of 0.97 and *SEP* of 140 mg/g. A similar result was obtained by Bureau *et al.* [60], who applied mid-infrared spectroscopy in the 6378-9900 nm range. The PLS model they established performed excellently, with *r_p_* of 0.98 and *RMSEP* of 9.0%. They also found phenolic compounds, contrary to sugar content and organic acid, could be affected by sample oxidation, grinding and storage temperature in a descending order of degree. The two researches proved the feasibility of predicting total polyphenol levels in apple using spectroscopic technology.

#### 3.1.5. Variety Discrimination

He *et al.* [48] applied Vis/NIR diffuse reflectance spectra to discriminate apple cultivars. Spectra of three cultivars including ‘Fuji’, ‘Red Delicious’ and ‘Copefrut Royal Gala’ in the wavelength range of 400–960 nm were obtained, processed with moving average and compressed using PCA. Based on the loading plots, wavebands of 650–690 nm and 550–565 nm were identified as being sensitive to varieties and used as input of a BP-ANN model. A discriminant accuracy of 100% could be achieved, with a residual error of 9.94 × 10^−5^. Moreover, wavelet transformation (WT) based on Daubechies 5 could reduce the size of variables to 4% [48]. Guo *et al.* [45] applied hyperspectral images in the wavelength range of 400–1000 nm for discrimination according to origins. Three CWs around 576, 678 and 971 nm were selected by PCA and texture analysis based on gray level co-occurrence matrix (GLCM) and used to build a SVM model, yielding a discriminant accuracy of 89.86% for the predicting sets. The excellent discriminant performance of CWs and wavebands indicated the bright future of online classification and instrument development. In the variety discrimination, spectroscopic and hyperspectral imaging techniques both show excellent performance with appropriate multivariate calibration techniques.

#### 3.1.6. Bruise Detection

Luo *et al.* [76] selected characteristic wavelengths (CWs) in the 380–1000 nm range for bruise detection. Each CW was considered as an independent classifier for bruise/normal identification and evaluated with receiver operating characteristic (ROC) analysis. The performance of the model based on CWs was compared with the PLS-DA model based on the full spectrum. The accuracies of both methods could exceed 95%. Similar accuracies was obtained by Baranowski *et al.* [44], who detected early bruises on six apple cultivars using hyperspectral imaging in the Vis/NIR region (400–2500 nm) and thermal imaging of emitted radiation in mid-wavelength infrared range (MWIR, 3500–5000 nm). The whole spectral range (400–5000 nm) was found useful. Minimum noise fraction (MNF) could yield accuracy rates of 87%–97%, better than PCA. The performance of linear discriminant analysis LDA, SVM and SIMCA were compared and the best one to distinguish bruised and intact apples was LDA, with a total success rate of 95%, while the best result for distinguishing deep and shallow bruised areas was obtained by SVM, with a total success rate of 77%. Huang *et al.* [77,78] employed two hyperspectral imaging systems in the 400–1000 nm and 1000–2500 nm range, respectively. PCA was used and CWs were determined based on the weighting coefficients plot of the best PC images. An overall classification accuracy of 90% and 97% could be obtained by the two systems, respectively.

These research works proved the feasibility of bruise discrimination based on spectra or hyperspectral images in the whole wavelength as well as characteristic variables selected, indicating the potential of instrument development and online detection. Although good performances were obtained based on spectroscopic and hyperspectral imaging techniques for bruise detection in the NIR region, subtle bruises could not be easily detected. The surface morphology and skin coloration can significantly affect the performance of bruise detection. Thus a chemometric-based hyperspectral imaging system is a good choice and is more appropriate for online applications. 

#### 3.1.7. Pigment

Pigment content was proved to link with quality attributes such as SSC and firmness. Zude *et al.* [79] found strong correlations between the peak absorbance of chlorophyll at 680 nm and harvest date (*r* = 0.59), background color (*r* = 0.74) and the starch index SI (*r* = 0.64). It also had some correlation with firmness (*r* = 0.48) and SSC (*r* = 0.46). Rutkowski *et al.* [80] found the index of anthocyanin (NAI), calculated as (I_780_I_570_)/(I_780_ + I_570_) significantly correlated with fruit firmness (*r* = 0.86) and titratable acid (*r* = 0.81) in ‘Golden Delicious’ apples. They claimed that NAI was the most suitable index to assess apple maturity, whereas Kuckenberg *et al.* [81] claimed NDVI better. Combined with further investigation, it could be concluded that the most suitable index representing maturity of apple fruit was variety-specific.

#### 3.1.8. Other Parameters

There are also some other attributes affecting the quality of apple that can be measured spectroscopically. Pissard *et al.* [6] found that vitamin C varied greatly among different cultivars. An overall model could just provide a coarse prediction (*r* = 0.89).

**Table 3:** Overview of applications for measuring quality attributes in oranges.

Quality Attribute	Cultivar	Method	Spectral Mode	Spectral Range/nm	Calibration Method	*r_p_*	*RMSEP*	Ref.
SSC or sugar content	GN	Spectroscopy	Reflectance	361–2488	PLS	0.93	0.59	[58]
	GN	Spectroscopy	Transmittance	1100–2500	PLS	0.90	0.46	[59]
	GN	Spectroscopy	Reflectance	820–950	LS-SVM	0.92	0.32	[62]
	Mixed	Spectroscopy	Reflectance	350–1800	PCA-BPNN	0.90	0.70	[63]
	Mixed	Spectroscopy	Reflectance	500–2300	PLS	0.91	0.74	[82]
				1100–2300		0.89	0.68	
	GN	Spectroscopy	Reflectance	350–1800	PCA-BPNN	0.90	0.68	[14]
	GN	Spectroscopy	Transmittance	350–1000	CARS-PLS	0.92	0.39%	[8]
	GN	Spectroscopy	Transmittance	465–1150	PLS	0.88	0.49%	[83]
	ST	Spectroscopy	Reflectance	400–1000	PCA-PLS	0.84	0.29 (*SEP*)	[15]
	HY					0.87	0.30 (*SEP*)	
	GN	Spectroscopy	Reflectance	450–1750	Spline-PLS	0.87	0.47	[18]
	GN	Spectroscopy	Reflectance	700–934	LS-SVM	0.85	0.41	[84]
Acidity	GN	Spectroscopy	Transmittance	1100–2500	PLS	0.64	0.70	[59]
						0.65 (pH)	0.13	
	Mixed	Spectroscopy	Reflectance	500–2300	PLS	0.83	0.17	[82]
						0.88 (pH)	0.15	
				1100–2300		0.77	0.19	
						0.81 (pH)	0.16	
Vitamin C	Mixed	Spectroscopy	Reflectance	1333–1835	PLS	0.96	0.039	[20,21]

GN: Gannan navel orange; HM: Hamlin orange; ST: Shatangju; HY: Huangyanbendizao.

### 3.2. Oranges

Rich in Vitamin C and other nutrients, orange is another widespread and popular fruit. Due to its short maturity period, the supply and demand contradiction is sharp. To extend the sales period, better storage and preservation methods are required as the internal quality of oranges declines during storage. If there wasn’t a rapid and simple approach to monitor their inner attributes, lots of oranges would decay and lose value. Most of the studies on the quality assessment of oranges have focused on attributes affecting its taste, including sugar content, acidity and Vitamin C. A brief overview is presented in Table 3.

#### 3.2.1. SSC or Sugar Content

Liu *et al.* [58] applied Vis/NIR diffuse reflectance spectroscopy to predict SSC of navel oranges. PLS models were built with 2nd derivative spectra in four different wavebands strongly correlated with SSC, *i.e.*, 361–2488 nm, 530–690 nm, 940–1420 nm and 1630–2488 nm. The model based on 940–1420 nm spectra provided a good result, with *r_p_* of 0.90 and *RMSEP* of 0.75, quite close to that obtained by the model based on the full spectrum. The *r_p_* of the PLS and PCR models was 0.93 and 0.61, respectively, with *RMSEP* of 0.59 and 0.69. The superiority of PLS to PCR was confirmed by Lu *et al.* [59], while Liu *et al.* [62] found that LS-SVM outperformed PLS. This result was in accordance with Sun *et al.* [84]. In the experiment of Liu *et al.* [18], the best performance was achieved by Spline-PLS. Cayuela and Weiland [82] compared 500–2300 nm and 1100–2300 nm spectra. The 600–750 nm spectra were excluded as they were strongly affected by skin chlorophyll, whose absorbance band corresponds to 680 nm. The *r_cv_* yielded was 0.91 and 0.89 respectively, with *RMSEP* of 0.74 and 0.68. Shao *et al.* [15] also found the 970–990 nm waveband to be particularly important while the 750–800 nm waveband made rather small contributions.

Moreover, Liu *et al.* [63] extracted characteristic wavelengths (CWs) by PCA, and used them as input of an ANN, yielding an *r_p_* of 0.90 and a *RMSEP* of 0.70. The result was quite similar with that reported by Liu *et al.* [14], who utilized Vis/NIR diffuse reflectance spectra in the 350–1800 nm range. PLS and back propagation neural network based on PCA (PCA-BPNN) were compared and the best result was achieved by the PCA-BPNN model combined with MSC, with *r_p_* of 0.90 and *RMSEP* of 0.68°Brix. In the study of Sun *et al.* [8], several variable selection methods including competitive adaptive reweighted sampling (CARS), uninformative variables elimination (UVE) and SPA were compared. The best result was achieved by the CARS-PLS model, with *r_p_* of 0.92 and *RMSEP* of 0.39%.

Xu *et al.* [83] investigated the influence of placement position. According to the different angles between incident light and the line composed by orange stem and pit, spectra were obtained at three different positions: vertical (90°), parallel (0°) and random. The best result was yielded by the model built with vertical spectra, with *r_p_* of 0.88, and *RMSEP* of 0.49%. Sun *et al.* [84] investigated the effect of three different reference points *i.e.*, λ_cv_(max), λ_cv_(min) and λ_cv_(median), which were identified by the coefficients of variation (CVs) at different wavelengths. The best result was achieved when λ_cv_(max) was used.

Taken together these results indicate that SSC or sugar content in oranges could be excellently predicted using spectroscopy. Spectra in the NIR range were particularly important for the prediction while spectra in the visible region could enhance the accuracy. Meanwhile, with regard to measurement modes, transmittance has slightly higher predictive outcomes than reflectance and interactance. Reflectance is the easiest mode to obtain measurements due to the relatively high light levels. LS-SVM was proved slightly better than PLS and PCA.

#### 3.2.2. Acidity

Lu *et al.* [59] employed Vis/NIR transmittance spectra to predict titratable acidity and available acidity (pH) in ‘Gannan’ navel oranges. PLS models outperformed PCR ones, but only coarse prediction results were obtained, with *r_p_* of 0.64 and 0.65 and *RMSEP* of 0.70 and 0.13, respectively. Cayuela and Weiland [82] got slightly better results with PLS models based on reflectance spectra in the 1100–2300 nm range. For pH, *r_cv_* was 0.81, while *RMSEP* was 0.16. For titratable acidity, *r_cv_* was 0.77 while *RMSEP* was 0.19. Better results were obtained using spectra in the 500–2300 nm range, with *r_cv_* of 0.88 and 0.83, respectively, indicating the possibility of acidity measurement using Vis/NIR spectroscopy. More efforts should be made to enhance the prediction performance, because total acidity prediction by NIRS has been considered difficult to achieve, due to the relatively low levels of organic acids found in oranges.

#### 3.2.3. Vitamin C

Xia *et al.* [20,21] predicted vitamin C content in oranges using spectra in the 833–2500 nm wavelength range. Several preprocessing algorithms were compared, including constant offset elimination (COE), vector normalization (VN), MSC, first and second derivative and Daubechies 3 WT with different decomposing levels. Daubechies 3 WT at level 4 was proved to be the best. The optimal waveband for prediction was 1333–1835 nm, and a PLS model built with spectra in this waveband performed excellent, with *r_p_* of 0.96 and *RMSECV* of 0.039 mg/g, indicating the feasibility of NIR spectroscopy for this application.

#### 3.2.4. Variety Discrimination

Shao *et al.* [15] employed diffuse reflectance spectra in the 400–1000 nm region to discriminate four cultivars, including ‘Shatangju’, ‘Huangyanbendizao’, ‘Gongju’ and ‘Huangdigan’. By using the predicted error sum of squares (PRESS) as an indicator, Daubechies 1WT with a decomposition length of 5 was chosen. Afterward, a BP-ANN model was established and yielded a discriminant accuracy of 100%, with a residual error of 8.27 × 10^−5^. Cen *et al.* [39] got a result at the same level using reflectance spectra in the 325–1075 nm wavelength range. The combination of BP-ANN and PCA yielded an accuracy of 100%, with *r_p_* of 0.998 and *RMSEP* of 0.18. Hao *et al.* [43] compared the discrimination performance of SIMCA and PLSDA based on diffuse reflectance spectra in the 350–1800 nm range. Both methods could reach a discriminant rate of 100% for all of the four cultivars. These results illustrated that orange varieties could be precisely classified using Vis/NIR spectroscopy technology.

#### 3.2.5. Other Parameters

Deng *et al.* [85] found that reflectance spectrum at 988 nm significantly correlated with SSC (*r* = 0.387**), SSC/acid ratio (*r* = 0.440**) and vitamin C (*r* = 0.309*). Both SSC and vitamin C were positively correlated with second derivative reflectance spectrum at 943 nm, with *r* of 0.339* and 0.355*, respectively. Cayuela and Weiland [82] measured some other quality attributes of orange including maturity index, firmness, juiciness, and fruit weight. Good results were acquired for most of these parameters, with *r_cv_* of 0.66–0.96 and *RPD* of 1.31–4.76.

### 3.3. Kiwifruit

Kiwifruit is a popular fruit with important medicinal and edible value. As a kind of climacteric variant fruit, the flavor and texture of kiwifruit change over time, so monitoring its internal quality rapidly and nondestructively is of great significance. Aside from indicators commonly used for assessing fruit quality, like sugar content, acidity and dry matter content, the firmness of kiwifruit could greatly affect its consumer acceptance. A number of investigations have been done. A brief review is presented in Table 4.

**Table 4:** Overview of applications for measuring quality attributes in kiwifruit.

Quality Attribute	Cultivar	Method	Spectral Mode	Spectral Range/nm	Calibration Method	*r_p_*	*RMSEP*	Ref.
SSC or sugar content	AD	Imaging spectroscopy	Reflectance	650–1100	PLS	0.92	1.18	[2]
	AD	Spectroscopy	Interactance	800–1100	PLS	0.95	0.39	[86]
	HW	Spectroscopy	Transmittance	400–1000	PLS	0.93	0.26	[87]
	AC	Spectroscopy	Interactance	520–1100	PLS	0.96	0.80 (*SEP*)	[88]
	Various	Spectroscopy	Interactance	800–1000	PLS	0.97	0.32%	[89]
	YF	Spectroscopy	Interactance	300–1140	PLS	0.96	0.31% (*SEP*)	[90]
	HW	Spectroscopy	Reflectance	800–2500	PLS	Unknown	0.68 (*SEP*)	[91]
	AD	Spectroscopy	Reflectance	408–2492	PLS	0.99	0.49	[92]
Acidity	HW	Spectroscopy	Transmittance	400–1000	PLS	0.94	0.076	[87]
	AD	Spectroscopy	Reflectance	408–2492	PLS	0.95	0.28% (*SEP*)	[92]
Firmness	AD	Spectroscopy	Interactance	800–1100	PLS	0.87	7.0	[86]
	AD	Spectroscopy	Reflectance	408–2492	PLS	0.94	3.32	[92]
	HY	Spectroscopy	Reflectance	833–2500	PLS	0.85	1.89	[19]
	ZH	Spectroscopy	Reflectance	1000–2500	NAP-PLS	0.88	0.88	[27]
Dry matter	AD	Spectroscopy	Interactance	800–1100	PLS	0.95	0.42%	[86]
	Various	Spectroscopy	Interactance	800–1000	PLS	0.97	0.29%	[89]
	YF	Spectroscopy	Interactance	300–1140	PLS	0.98	0.24% (*SEP*)	[90]
	ZH	Spectroscopy	Reflectance	1000–2500	siPLS	0.90	0.53%	[34]

AD: *Actinidia deliciosa*; HW: Hayward; AC: *Actinidia chinensis*; YF: yellow-fleshed kiwifruit; ZH: Zhonghua; HY: Huayou.

#### 3.3.1. SSC

McGlone and Kawano [86] employed interactance spectra to predict the SSC of kiwifruit in the 800–1100 nm wavelength range. A PLS model built with 2nd derivative spectra showed excellent performance, with *r* of 0.95 and *RMSEP* of 0.39°Brix. The result was much better than those reported by Martinsen and Schaare [2] and Moghimi *et al.* [87], who used reflectance and transmittance spectra, respectively. The superiority of interactance spectra was confirmed by Schaare and Fraser [88]. Lee *et al.* [92] predicted SSC with a broad spectral range (408–2492 nm), and an excellent result was observed (*r_p_* = 0.99; *SEP* = 0.49°Brix). McGlone *et al.* [89] found the 800–1000 nm waveband performed well. They also proved the feasibility of using data obtained from unripe kiwifruit to predict SSC of ripe fruit, with *r* of 0.96 and *RMSEP* of 0.39%. A similar investigation was done by McGlone *et al.* [90], who proved that the SSC prediction model based on post-storage spectra was better than that based on harvest-time spectra. A possible explanation was that Vis/NIR spectroscopy was better at predicting total carbohydrate concentration, which consists of starch and soluble sugar in about the same amounts at harvest time but mainly of soluble sugar after storage. This was supported by the observation that predicting SSC of post-storage kiwifruits by using the model built with harvest-time spectra could yield more accurate results (*SEP* = ±0.38%). Arazuri *et al.* [91] investigated the influence of temperature. Reflectance spectra in the 800–2500 nm range were obtained at three different sample temperatures (0.5, 10 and 20 °C) and the best performance was achieved using spectra obtained under 0.5 °C, with *SEP* of 0.68.

According to these studies, all three spectral modes provide good accurate estimates of SSC. However, interactance spectra were better for predicting SSC in kiwifruits, and transmittance mode was better than reflectance mode. A possible explanation was that interactance spectra were less susceptible to specular reflections, which was probably the source of larger errors. Spectra in the 800–1000 nm range were very important, while a slightly better result could be achieved by using spectra in the whole Vis/NIR range. The ripening stage of kiwifruit affects greatly the accuracy of SSC prediction and accurate prediction was based on ripe kiwifruit.

#### 3.3.2. Acidity

Moghimi *et al.* [87] predicted the acidity in kiwifruits with transmittance spectra in the 400–1000 nm range. A PLS model based on spectra processed with SNV, median filter and 1st derivative could yield a result with *r_p_* of 0.94 and *RMSEP* of 0.076, close to the values obtained by Lee *et al.* [92], who used spectra in the 408-2492 nm range and predicted acidity with *r_p_* of 0.95 and *SEP* of 0.28%. The feasibility of Vis/NIR spectroscopy for predicting acidity in kiwifruit was thus proved.

#### 3.3.3. Firmness

McGlone and Kawano [86] employed interactance spectra in the 800–1100 nm wavelength range to assess the firmness of kiwifruit. The performance of the PLS model was barely satisfactory, with *r* of 0.81 and *RMSEP* of 7.8 N. A better result could be obtained when the samples were sorted in terms of origins and sizes (*r* = 0.87; *RMSEP* = 7.0 N). However the model performed poorly against independent data sets, indicating the existence of secondary correlations due to fruit characteristics, which were not directly related to fruit firmness. Liu *et al.* [19] used diffuse reflectance spectra in the 833–2500 nm wavelength range. Both first and second derivative could improve the prediction accuracy, while SNV and MSC could not. The PLS model based on first derivative spectra could yield the optimal result, with *r_p_* of 0.85 and *RMSEP* of 1.89. A better result was reported by Lee *et al.* [92], who used spectra in the 408–2492 nm range. Flesh firmness was predicted with *r_p_* of 0.94 and *SEP* of 3.32 N. Lv *et al.* [25] optimized modeling wavelengths and decreased the number of principal components (PCs) with net analyte processing (NAP). Through NAP-PLS, an optimal model was established with five PCs selected in five wavebands (1862–1927, 2164–2198, 1605–1653, 1293–1429 and 1511–1600 nm) and predicted firmness with *r_p_* of 0.88 and *RMSEP* of 0.88. In all this research, the firmness of kiwifruit could only be coarsely predicted, suggesting that perhaps there is too little pectin in the kiwifruit for NIRS to pick up, so further research should be done to enhance the prediction accuracy.

#### 3.3.4. Dry Matter (DM)

McGlone and Kawano [86] employed a PLS model based on interactance spectra in a narrow waveband (800–1100 nm) to predict the DM of ‘*Actinidia deliciosa*’, yielding an *r_p_* of 0.95 and *RMSEP* of 0.42%. McGlone *et al.* [89] applied interactance spectra processed with S-G smoothing and area normalization sequentially. DM of unripe and ripe fruits could be accurately predicted based on 800–1000 nm spectra. The PLS model based on ripe fruits yielded an *r* of 0.97 and *RMSEP* of 0.29%. DM of the ripe kiwifruits could also be predicted with data obtained when the fruit is unripe, with *r_p_* of 0.97 and *RMSEP* of 0.39%. Lue *et al.* [34] investigated the potential of long-term DM prediction. They obtained spectra and dry matter contents of some unripe and ripe kiwifruits and extracted CWs with synergy interval partial least square (siPLS). A model based on spectra of unripe kiwifruits and DM of ripe fruits was built, with *r_p_* of 0.90 and *RMSEP* of 0.53%. McGlone *et al.* [90] and Feng *et al.* [93] also proved that DM in kiwifruit could be accurately predicted. In conclusion, these studies proved that dry matter in kiwifruits could be excellently predicted with NIR spectroscopy, and the NIR method gives a good predictive relationship with SCC by finding spectral information that is independent of the DM for kiwifruit. Interactance spectra were most commonly employed.

#### 3.3.5. Other Parameters

Schaare and Fraser [88] applied Vis/NIR spectroscopy in the reflectance, interactance and transmittance mode to measure density and flesh color of kiwifruit. Best performances were all achieved by a PLS model built with interactance spectra in the 520–1100 nm wavelength range. Density and flesh hue angle were predicted with *r* of 0.86 and 0.91 and *SEP* of 3.6kg/m^3^ and 1.6°, respectively. Tavakolian *et al.* [41] applied reflectance spectra in the 1130–2220 nm range for the classification of kiwifruit varieties with different post-harvest date. PCA was performed and showed that the first three PCs could explain 99% of the variance. SIMCA was applied based on three CWs at 1190, 1450 and 1940 nm. The total classification accuracy was 92.3%.

### 3.4. Peaches

Peaches are another kind of tasty and nutritious fruit. Like kiwifruit, the firmness of peaches changes greatly over time, thus firmness, along with sugar content and acidity, by which the flavor of peaches are mainly determined, attract the interest of most researchers. A brief review is presented in Table 5.

**Table 5:** Overview of applications for measuring quality attributes in peach.

Quality Attribute	Cultivar	Method	Spectral Mode	Spectral Range/nm	Calibration Method	*r_p_*	*SEP*	Ref.
SSC or sugar content	Various	Spectroscopy	Transmittance	800–1050	MLR	0.20–0.91	0.49%–1.63%	[51]
	Unknown	Spectroscopy	Transmittance	1000–2500	Stepwise MLR	0.53	Unknown	[94]
	Mixed	Spectroscopy	Reflectance	325–1075	ICA-LS-SVM	0.95	0.42 (*RMSEP*)	[36]
	Unknown	Multispectral scattering		632, 650, 670, 900	MLR	0.97	0.69	[3]
	SH	Spectroscopy	Interactance	870, 878, 889, 906	MLR	0.97	0.50	[95]
	Mixed	Spectroscopy	Reflectance	1279–2331	PLS	0.96	0.57	[72]
	DB	Spectroscopy	Reflectance	800–2500	PLS	0.94 (*r_cv_*)	0.57 (*RMSECV*)	[96]
Acidity	Mixed	Spectroscopy	Reflectance	928–2331	PLS	0.95	0.13	[96]
	Mixed	Spectroscopy	Reflectance	325–1075	ICA-LS-SVM	0.96	0.047 (*RMSEP*)	[36]
Firmness	Unknown	Multispectral scattering		670, 780, 850, 900	MLR	0.95	1.56	[3]
	Mixed	Multispectral scattering		680, 880, 905, 940	MLR	0.82	18.55	[97]
	RH	Hyperspectral scattering		500–1000	MLR	0.88	14.2	[98]
	CS	Hyperspectral scattering				0.76	19.1	
	White peach	Spectroscopy	Reflectance	800–2500	PLS	0.89	5.42 (*RMSEP*)	[99]

SH: Shimizu Hakuto; DB: Dabaitao; RH: Red Haven; CS: Coral Star.

#### 3.4.1. SSC or Sugar Content

Peiris *et al.* [51] and Jiang *et al.* [94] applied transmittance spectroscopy to predict SSC. Neither of the results was satisfactory. Kawano *et al.* [95] got better result using interactance spectra. In 2nd derivative spectra, clear differences among peaches with different Brix values had been observed at the wavelength of 906 nm, which was assigned to sucrose. The best prediction result was yielded by the linear regression model built with spectra at 870, 878, 889 and 906 nm, with *r* of 0.97 and *SEP* of 0.50°Brix. This result was in the same level as those of Liu *et al.* [72] and Ma *et al.* [96], both of which employed reflectance spectra in the NIR range. The former one reported the first derivative spectra yielded the best result with *r_p_* of 0.96 and *SEP* of 0.54, while the latter one found the original spectra more suitable, with *r_cv_* of 0.94 and *RMSECV* of 0.57. Shao *et al.* [36] applied independent component analysis (ICA) and latent variables analysis (LVA) to CWs from reflectance spectra in the wavelength range of 325–1075 nm. PLS and LS-SVM models were built with the CWs selected. LS-SVM always performed better than PLS. The optimal result was achieved by the ICA-LS-SVM model, with *r_p_* of 0.95 and *RMSEP* of 0.42. Liu *et al.* [3] obtained spectral scattering profiles at wavelengths of 632, 650, 670, 780, 850 and 900 nm and fitted them with Lorentzian distribution with three parameters. MLR models were established to relate SSC with Lorentzian parameters based on different number of wavelengths respectively. The optimal performance was achieved by the combination of spectral images at 632, 650, 670 and 900 nm, with *r* of 0.97 and *SEP* of 0.69°Brix.

Experiments showed that transmittance spectra contain little effective information related with SSC in peaches. We assumed that the incident radiation couldn’t permeate the peach core, which could be verified by the fact that when a similar system was utilized to assess SSC in apples, a much more accurate result was obtained [94]. Models built with selected CWs could yield results as good as those based on reflectance or interactance spectroscopy, indicating the feasibility of online detection.

#### 3.4.2. Acidity

Liu *et al.* [72] used FT-NIR reflectance spectroscopy in the 928–2331 nm waveband to measure the valid acidity (pH) in peach. The PLS model could obtain a result with *r_p_* of 0.95 and *SEP* of 0.13. Shao *et al.* [36] got a similar result (*r_p_* = 0.96; *RMSEP* = 0.047) from a LS-SVM model built with CWs selected by ICA. The feasibility of spectroscopy was thus verified, but the low acid content in the peaches might cause relative insensitivity for prediction valid of acidity.

#### 3.4.3. Firmness

Lu and Peng [97] predicted peach firmness using spectral scattering profiles at wavelengths of 680, 880, 905 and 940 nm. Soft peaches were found to have broader scattering profiles than firm ones, especially at 680 nm. A Lorentzian distribution function with three parameters was used to fit the scattering profiles with a mean *r*^2^ of above 0.998. MLR was employed to relate Lorentzian parameters with firmness. When models were built with peaches from different orchards, respectively, and the optimal result was acquired with *r_p_* of 0.87 and *SEP* of 14.57 N. When a model was established with samples from two orchards, a lower *r_p_* (0.82) and a higher *SEP* (18.55 N) was obtained. Liu *et al.* [3] got a better result based on the combination of scattering profiles at 670, 780, 850 and 900 nm (*r* = 0.95; *SEP* = 1.56 N). Moreover, Lu and Peng [98] utilized hyperspectral scattering profiles of 500–1000 nm to assess the firmness of two cultivars: ‘Red Haven’ and ‘Coral Star’. The profiles were fitted using a Lorentzian distribution function with two parameters, with a mean *r*^2^ above 0.99. Then MLR models were established to relate Lorentzian parameters and their contributions at different wavelengths with peach fruit firmness. The highest correlation among all individual wavelengths was found at the wavelength 677 nm, which corresponds to chlorophyll absorption. When two Lorentzian parameters *a* and *b* (*a* represented the peak scattering value while *b* the full scattering width at one half of the peak value) were used as independent variables, optimal results were achieved for the two cultivars with *r* of 0.88 and 0.76 respectively.

Fu *et al.* [99] investigated the anisotropy of firmness and spectra, regarding to measuring spots at different latitudes and longitudes. Both spectral absorbance and firmness of peaches were proved to be affected by longitudes and latitudes. The collaboration of spectra from different latitude and longitude, and proper pretreatment methods like scattering correction or derivative could improve the prediction. The best performance was achieved by the holistic model built with spectra processed by MSC (*r* = 0.89; *RMSEP* = 5.42 N).

These studies above proved that spectral scattering and hyperspectral scattering were feasible for predicting firmness of peach. NIRS does not provide quantitative information on light scattering in peach, which leads to its capability for predicting structurally firmness limited or difficult to justify, and both chlorophyll and water status have an important effect on firmness. More efforts should be made to enhance the prediction accuracy, such as by utilizing hyperspectral scattering.

#### 3.4.4. Variety Discrimination

Wu *et al.* [38] applied reflectance spectra in the 401–1000 nm wavebands for the variety discrimination of three peach cultivars *i.e.*, ‘Mengyin’, ‘Fenghua’ and ‘Jinhua’. Eight PCs were selected by PCA to build a SDA model, which could yield a discrimination accuracy of 100%, better than that of the PLS model. A similar result was obtained by Li *et al.* [100], who used spectra in the same wavelength range to classify the cultivars ‘Milu’, ‘Hongxianjiu’ and ‘Dabaitao’. Multiple discriminant analysis (MDA) based on the first eight PCs yielded a recognition rate of 100%. Fu *et al.* [99] compared the discriminant accuracy of discriminant analysis (DA), SIMCA and discriminant PLS (DPLS) using diffuse reflectance spectra in the region of 800–2500 nm. The discriminant accuracy of both DA and SIMCA were above 92%, while that of DPLS was slightly better, reaching 95%. To conclude, Vis/NIR spectroscopy was proved feasible for peach variety discrimination. The full wavelength was useful while spectra in the visible range were essential.

#### 3.4.5. Other Parameters

Zwiggelaar *et al.* [101] combined spectroscopy and machine vision to detect bruises on peaches. After the samples were bruised, they were divided into three groups, one of which was left at room temperature while other two were put in a cold conditions for one week and two weeks, respectively, to inhibit ripening. Reflectance spectra at bruised and un-bruised areas and spectral images at 930 and 970 nm were obtained. Results indicate a success rate of only 65%, perhaps indicating a wrong wavelength choice. More studies should be done to improve the success rate. Takano *et al.* [102] evaluated polyphenols in peach using NIR spectroscopy in the 1100–2500 nm wavelength range. A coarse prediction could be achieved, with *r* of 0.80 and *SEP* of 14.7 mg/100 g. By multiple regression analysis, spectra at 1720 nm were proved to have the highest correlation with polyphenol content. 

To classify peaches according to their maturity, Lleó *et al.* [103] compared two multispectral classification methods based on red images (R) and a combination of R and infrared images (R/IR) respectively. The spectral images were obtained by three CCD cameras (450, 675 and 800 nm). The R/IR method performed better as it eliminated the effect of fruit shape on light reflectance. Reflectance at 680 nm (chlorophyll absorption peak) increased, while firmness decreased during the ripening process.

### 3.5. Strawberries

With their attractive appearance, luscious taste and rich nutritional value, strawberryies have won the affection of people all over the world. Due to their soft tissues and high moisture content, strawberries are quite perishable, so more effective monitoring and detection methods are required. Being the sugar content, acidity and firmness the most important attributes that could affect quality and price, these draw the attention of most researchers.

#### 3.5.1. SSC

ElMasry *et al.* [52] used hyperspectral imaging in the Vis/NIR region (400–1000 nm) to predict the SSC in strawberry. Some samples were kept in room temperature for several days while others were kept under 5 °C conditions to guarantee wide variations of internal properties. The PLS model built with spectra processed mean-centering and automatic baseline correction performed well, with *r_p_* of 0.85 and *SEP* of 0.184. A MLR model established with CWs selected by β-coefficients of PLS achieved a close result, with *r_p_* of 0.80 and *SEP* of 0.211. A similar result was obtained by Sanchez *et al.* [11], who built a modified PLS model with reflectance spectra in the 1600–2400 nm wavelength range (*r* = 0.89; *RPD* = 2.15). Nishizawa *et al.* [104] reported better results using spectra in the 700–925 nm range (*r* = 0.93; *SEP* = 0.9%). Pretreatment methods including 2nd derivative and MSC were proved useless for improving the accuracy. The best result was reported by Guo *et al.* [35], who employed a synergy interval PLS (siPLS) model based on 1st derivative spectra in the 833–2631 nm wavelength range (*r_p_* = 0.97; *RMSEP* = 0.29).

Shi *et al.* [37] attempted to simplify the prediction. CWs were selected using backward interval PLS (BiPLS) combined with simulated annealing algorithm (SAA). Spectra in the 1000–2500 nm wavelength range were divided into 21 subsets and characteristic subsets were determined by BiPLS. Then SAA was applied to select CWs in these informative regions. Finally seven CWs, in the 1135–1322 nm range were selected to build a MLR model. The predictive performance of this BiPLS-SAA-MLR model was better than those of the PLS and BiPLS models, with *r_p_* of 0.94 and *RMSEP* of 0.43. From these research works, the feasibility of the spectroscopic method for SSC prediction in strawberries were verified. Models built with CWs could yield very good results, indicating the potential for instrument development and online detection.

#### 3.5.2. Acidity

Shao *et al.* [22] applied reflectance spectroscopy in the 400–1000 nm range to predict the acidity of strawberries. After some defective spectra were eliminated using PCA, a PLS model was built, with *r_p_* of 0.92, *SEP* of 0.027 and *RMSEP* of 0.026. After wavelet transform (WT) was applied to compress the spectral data, a PLS model was established with *r_p_* of 0.86, and *RMSEP* of 0.026. A similar result was observed by ElMasry *et al.* [52], who applied hyperspectral imaging in the 400–1000 nm range to assess the pH. The PLS model yielded an *r_p_* of 0.87 and *SEP* of 0.13, while the MLR model achieved better results, with *r_p_* of 0.94 and *SEP* of 0.091. Sanchez *et al.* [11] applied reflectance spectra in the 1600–2400 nm wavelength range. The model was built with modified PLS regression. For the prediction of titratable acidity, an acceptable result was obtained, with *r* of 0.73 and *RPD* of 1.43, but the predictive performance for pH was not so good (*r* = 0.48), probably indicating an unsuitable wavelength choice. Although the feasibility of the spectroscopy method for predicting acidity in strawberries were confirmed and the ripening stage did not seriously affect pH prediction, further investigations should be done to figure this out.

#### 3.5.3. Firmness

Tallada *et al.* [66] assessed firmness in strawberries using hyperspectral images in the 650–1000 nm range. A stepwise MLR model based on three optimal wavelengths of 685, 865 and 985 nm could give a result with *r_p_* of 0.79 and *SEP* of 0.35 MPa. The performance of a modified PLS model based on reflectance spectra in the 1600–2400 nm range was not satisfactory either, with *r* of 0.66 and *RPD* of 1.35 [11]. As well as SSC and acidity, firmness prediction is feasible, which is confirmed by the NIRS. Further research should be done to enhance the prediction accuracy.

#### 3.5.4. Variety Discrimination

Niu *et al.* [50] used spectra ranging from 1100 to 2200 nm for the classification of varieties ‘Tianbao’, ‘Fengxiang’ and ‘Mingxing’. The performance of BP-ANN, LS-SVM and discriminant analysis (DA) was compared and the best result was achieved by the BP-ANN model, with a total discrimination rate of 97.14%. Yan *et al.* [105] tried to simplify the discrimination. Spectra of three varieties were obtained in the 350–2500 nm range. 2nd derivative combined with SNV and moving average was ascertained as the best data pretreatment method. CWs were determined as 548–562 nm by the correlation coefficient and threshold value method. The optimal predictive performance was achieved by the PLS-ANN model, with *r_p_* of 0.97 and *RMSEP* of 0.46, slightly better than those of the PLS and PCR models. However, the discriminant accuracy reported by Sanchez *et al.* [11], who built a PLS-DA model with reflectance spectra in the 1600–2400 nm wavelength range was only 63%, raising some uncertainty as to the feasibility of Vis/NIR spectroscopy for discriminating strawberry varieties.

#### 3.5.5. Other Parameters

Nagata *et al.* [106] investigated the feasibility of NIR hyperspectral imaging in the wavelength range of 650–1000 nm for detecting compression bruises on strawberries. Hyperspectral images of strawberries subjected to different levels of bruising force were obtained during 0–4 days after bruising. Three discriminant methods including linear discriminant analysis (LDA), normalized difference and artificial neural network (ANN) performed equally well. Through stepwise LDA, two optimal wavelengths *i.e.*, 825 and 980 nm were identified and the classification efficiency for bruised and non-bruised pixels could reach 86.5% and 99.7%, respectively. Detected bruises were found to significantly decrease along storage time. 

Sanchez *et al.* [11] studied bulk skin or external color values of strawberries including L*, a* and b*. Chroma and hue angle could be calculated with these parameters. The prediction results for L*, a* and chroma were acceptable, with *r* of 0.77 and *RPD* of 1.56 for all the three parameters, while for b* and hue angle, the result was not good (*r* ≤ 0.44), which suggested that NIRS prediction for b* and h* was not feasible.

### 3.6. Grapes

Not only a tasty fruit but also an ingredient for brewing wine, grape is an important economic crop cultivated all over the world. It is the sugar content and acidity in grapes that determine their flavor and indicate the ripeness, thus attracting the attention of researchers. It has been proved that grapes contain some precious nutritional ingredients including anthocyanins and polyphenols, so some work has also been done for measuring these ingredients.

#### 3.6.1. SSC or Sugar Content

Fernandez-Novales *et al.* [107] observed excellent results using transmittance spectra in the 700–1060 nm wavelength range. The PLS model based on spectra processed with Norris first derivative performed excellently, with *r_cv_* of 0.99 and *RMSECV* of 0.46. Herrera *et al.* [108] evaluated the feasibility of diffuse transmittance and interactance spectra for SSC prediction. Two spectral regions *i.e.*, 750–1100 and 650–1100 nm, were used to establish PLS models to predict SSC in three grape cultivars: ‘Cabernet Sauvignon’, ‘Chardonnay’ and ‘Carmenere’. The optimal performance for all cultivars was achieved by models based on 650–1100 nm spectra, with *r_p_* above 0.90 and *RMSEP* lower than 1.2. Transmittance spectra performed slightly better compared to interactance ones. Pretreatment methods had no significant effect, as confirmed by Larrain *et al.* [109], who applied reflectance spectra in the 640–1100 nm range to predict sugar content. The prediction performance of the PLS models they established was excellent, with *r_p_* of 0.93–0.96 and *RMSEP* of 1.01–1.27 for different cultivars. 

Wu *et al.* [110] simplified the prediction with BP-ANN. Vis/NIR diffuse reflectance spectra in the 400–1000 nm waveband were obtained. Three principal factors were identified by PLS based on the reliabilities, then the scores of the three selected principal factors were used as the input of a three-layer BP-ANN model. Its prediction accuracy outperformed the PLS model, with *r* of 0.95 and *RMSEP* of 0.11. Fernandez-Novales *et al.* [29] selected four CWs including 909, 951, 961 and 975 nm by the loading weights of latent variables and used them to build a MLR model for sugar content prediction. A satisfactory result could be obtained, with *r* of 0.96, *SEP* of 20.0 g/L and *RMSEP* of 20.5 g/L. The CWs they selected were not the same as those identified by Cao *et al.* [30], who applied Vis/NIR reflectance spectra in the 400–1000 nm wavelength range. A genetic algorithm was executed and five wavelengths *i.e.*, 418, 525, 556, 633 and 643 nm were identified as CWs. A PLS model was built for prediction, with *r_p_* of 0.91 and *RMSEP* of 0.93. Omar [69] built a MLR model with spectra of 605, 729, 830, 910 and 950 nm (*r* = 0.97, *RMSE* = 0.18°Brix).

Taking all these studies together, the feasibility of Vis/NIR spectroscopy was verified for SSC prediction. NIRS was sensitive to sugar content changes during the different ripening stages. Spectra in the 400–1000 nm range could yield excellent prediction results and transmittance spectra performed slightly better than interactance spectra.

#### 3.6.2. Acidity

Larrain *et al.* [109] proved that the pH of different grape cultivars could be predicted with *r_p_* of 0.75–0.89 and *RMSEP* of 0.088–0.16 using reflectance spectra ranging from 640 to 1100 nm. Cao *et al.* [30] obtained spectra of samples of three cultivars in the 400–1000 nm wavelength range. The performance of the GA-LS-SVM model was better than that of the PLS model, with *r_p_* of 0.98 and *RMSEP* of 0.13. A very close result was obtained by Omar [69], who found wavebands of 922–923 and 990–995 nm important. Their best result was achieved by a MLR model based on spectra of 605, 923 and 990 nm (*r* = 0.87; *RMSE* = 0.11).

However, Fernandez-Novales *et al.* [107] did a similar prediction for pH and tartaric acid using NIR transmittance spectra in the 700–1060 nm wavelength range and obtained less accurate results (*r_cv_* = 0.52, 0.41; *RMSECV* = 0.22, 2.02, respectively), indicating some uncertain difficulties involved in the prediction for this attribute in grapes, although there is still a high correlation with the reference pH sensor.

#### 3.6.3. Anthocyanin

Larrain *et al.* [109] evaluated anthocyanin concentration in different grape cultivars using spectra ranging from 640 to 1100 nm. PLS models yielded acceptable results (*r_p_* = 0.79–0.83) for most cultivars, except for Pinot Noir (*r_p_* = 0.63). This uncertainty was confirmed by Kemps *et al.* [68], who did similar research with reflectance spectra in the 320–1660 nm range. Prediction wasn’t feasible except for ‘Syrah’, in which anthocyanins could be predicted with *r_p_* of 0.8, making the method questionable.

#### 3.6.4. Variety Discrimination

Cao *et al.* [30] applied Vis/NIR reflectance spectroscopy in the 400–1000 nm wavelength range for the discrimination of three grape cultivars, namely ‘Manaizi’, ‘Mulage’ and ‘Heiti’. Firstly GA was applied to select CWs and four wavelengths *i.e.*, 636, 649, 693 and 732 nm were identified. Then a LS-SVM model was built, which could reach a total discrimination accuracy of 96.6%. For individual cultivars, the accuracy rate was 93.9% for ‘Manaizi’, 97.6% for ‘Mulage’ and 100% for ‘Heiti’. In another study [40], the combination of BP-ANN and PCA were applied. PCA was first applied for cluster analysis. The spectra of ‘Heiti’ were found significantly different from the other two cultivars especially in the 520–640 nm wavelength range. Then a BP-ANN model was established by using the first 10 PCs to discriminate the other two cultivars. A total discriminant accuracy of 98.3% could be achieved, slightly better than that yielded by SIMCA, which was 96.6%. A simplified BP-ANN model built with CWs of 452, 493, 542 and 668 nm could yield a very close accuracy rate of 97.4%, indicating the feasibility of online detection.

#### 3.6.5. Other Parameters

Fernandez-Novales *et al.* [107] estimated the maturity of grapes using a maturity index, calculated by dividing reducing sugar content by titratable acid content. This index could be predicted with *r_cv_* of 0.77 and *RMSECV* of 10.2. Considering the low accuracy obtained in the prediction of titratable acid, this result was inspiring and indicated a possibility of classifying grapes based on this index. Kemps *et al.* [68] reported that the concentration of polyphenols could not be predicted in any of the cultivars they used.

### 3.7. Jujube

Having been a kind of popular fruit in China for thousands of years, jujube is increasingly valued worldwide for its rich nutrients. Some studies in which spectroscopy and hyperspectral imaging technology are employed have been carried out for quality assessment of jujubes in recent years.

Zhang *et al.* [64] applied reflectance spectra in the 400–2400 nm range to predict SSC in three jujube cultivars. PCA was used on the spectra processed with smoothing and MSC. Six principal components were selected and employed as input of the BP-ANN model, which could predict SSC with a relative deviation lower than 10%. Wang *et al.* [46] compared reflectance, interactance and transmission spectra in the 310–1100 nm wavelength range for predicting SSC. Interactance spectra were proved to be the best choice, which yielded prediction results with *r_p_* of 0.74–0.91 and *RMSEP* of 2.0–3.2°Brix. The optimal performance was achieved by the model based on 2nd derivative spectra, with *r_p_* of 0.91. The fruit stone of jujubes affects the spectral characteristics of light reflected from or transmitted through the jujube. If each jujube fruit lacks a hard stone, transmission spectral measurements are effective at revealing the SSC. Zhang *et al.* [111] selected CWs for SSC prediction from reflectance spectra in the 350–2500 nm waveband using SPA and stepwise regression analysis (SRA). Wavelengths at 1374 nm and 1718 nm were identified by both methods, suggesting their importance. The best result was acquired by the PLS model based on whole spectra, with *r_p_* of 0.89 and *RMSEP* of 1.09. The LS-SVM model combined with SPA could yielded an acceptable result with *r_p_* of 0.80 and *RMSEP* of 1.40, better than that of the LS-SVM based on whole spectra. He *et al.* [65] employed hyperspectral images in the 900–1700 nm wavelength range for the prediction of SSC in jujubes. Five characteristic wavelengths were identified by PCA. The BP-ANN model yielded an *r_p_* of 0.90 and *RMSEP* of 1.98.

Wang *et al.* [112] tested the applicability of reflectance, interactance and transmittance spectroscopy in the 310–2150 nm wavelength range for detecting internal insect infestations in jujubes. Spectra were divided into three wavebands *i.e.*, 310–1000 nm (VSWNIR), 1000–2150 nm (LWNIR), and 310–2150 nm (Vis/NIR). The highest discriminant rates obtained were 90% for reflectance, 97% for transmittance and 100% for interactance. In the VSWNIR region, transmittance spectra yielded better performance while in the LWNIR region, interactance spectra were the most feasible. This was probably because light in the VSNIR range could transmit through the fruit core more effectively than that in the LWNIR range, leading to increased reflectance and decreased transmittance. Further, Wang *et al.* [47] compared the ability of the three modes of spectra for detecting internal insect infestation with different damage levels. Discriminant functions based on CWs were derived based on stepwise discriminant analysis (SDA). Result showed that reflectance and interactance spectra in the VSWNIR region could discriminate severely infested jujubes from slightly damaged ones the best. Wang *et al.* [113] applied hyperspectral imaging in the 400–720 nm wavelength range to detect external insect infestation. Three CWs *i.e.*, 690, 650 and 500 nm, which corresponded to chlorophyll a, chlorophyll b and carotenoids respectively, were selected by SDA. Over 98.0% of intact jujubes and 94.0% of insect-damaged jujubes could be correctly discriminated, achieving an overall discriminant accuracy of 97.0%. Both the internal and external infestations could be identified with an accuracy of above 97%, indicating the feasibility of Vis/NIR method.

Zhang *et al.* [111] applied NIR reflectance spectroscopy for the detection of subtle bruises on jujubes. Spectra of 350–2500 nm were acquired and processed with MSC. Nine wavelengths *i.e.*, 1869, 2128, 1430, 827, 359, 2477, 1357, 1643 and 762 nm were identified as CWs using SPA. Then four principal components (PCs) were identified from the CWs using PCA and performed as input to establish a LS-SVM model. This MSC-SPA-PCA-LS-SVM model yielded a discriminant accuracy of 100%.

### 3.8. Bananas

Tarkosova and Copikova [114] applied NIR spectroscopy in the 1100–2500 nm wavelength range to assess sugar content in bananas. Modified PLS models were established for prediction. The total sugar content could be predicted with *r_p_* of 0.99 and *SEP* of 0.80%, for sucrose, glucose and fructose, they could be predicted with *r_p_* of above 0.97 and *SEP* of 0.16%–0.78%.

Jaiswal *et al.* [53] predicted DM, pH, SSC and acid-Brix ratio (ABR) in bananas using transmittance spectra in the 299–1100 nm wavelength range. SSC could be best predicted by a PLS model built with original spectra in the 955–982 nm waveband (*r_p_* = 0.81) and pH could be best predicted by PLS model built with spectra processed with baseline correction in the 1009–1036 nm range (*r_p_* = 0.83). Though a higher *r_c_* could be obtained by MLR, there was a big gap between *r_c_* and *r_p_*, indicating unstability. For DM, the best result was achieved by the MLR model built with original spectra in the 1063–1089 nm wavelength range (*r_p_* = 0.83). As to ABR, the PLS model built with spectra processed by MSC combined with baseline in the 955–982 nm wavelength range yielded the best result (*r_p_* = 0.78).

Davey *et al.* [67] applied Vis/NIR reflectance spectroscopy in the 367–2388 nm wavelength range to measure total carotenoids, α-carotene, β-carotene, c-carotene and lutein in banana. PLS models were built based on 1st S-G derivative spectra. Results showed that total carotenoids and β-carotene could be measured accurately (*r_p_* = 0.98, 0.96; *RPD* = 3.34, 2.74, respectively). For α-carotene and c-carotene, results were acceptable with *r_p_* of 0.91 and 0.90 and *RPD* of 1.68 and 1.96, respectively. However, for lutein the result was not satisfactory, with *r_p_* of 0.75 and *RPD* of 1.16. Considering that 90% of the carotenoids in bananas were α-carotene and β-carotene, it was feasible to measure carotenoids in banana with Vis/NIR spectroscopy.

Subedi and Walsh [115] measured dry matter DM and SSC in banana mesocarps with transmittance spectroscopy in the 500–1050 nm wavelength range. The result obtained for DM was not good, probably due to the thickness of the peel. For SSC, excellent results were obtained from ripening and ripen banana mesocarps (*r_cv_* > 0.93; *RMSECV* < 0.80%). However, for green and over-ripe bananas prediction results were not satisfactory, indicating that mesocarp SSC was highly correlated with peel color.

### 3.9. Mangos

Jha *et al.* [116] applied reflectance spectroscopy to measure SSC and pH in seven mango cultivars. The optimal results were obtained by PLS models based on 2nd derivative spectra in the 1600–1799 nm range, with *r_p_* of 0.76 and 0.70 and *SEP* of 3.23 and 0.72, respectively. Although MLR models yielded higher *r_c_*, the gap between calibration and prediction indicated instability. Their results were inferior to those reported by Schmilovitch *et al.* [117] who applied NIR reflectance spectroscopy to predict firmness, SSC, acidity and storage period of mangos. Spectra were acquired in the 1200–2400 nm wavelength range. Best performances for predicting firmness, SSC, acidity were all achieved by MLR models built with 2nd derivative absorbance spectra, with *r_p_* of 0.91, 0.96, 0.78 and 0.97 and *SEP* of 17.14, 1.223, 0.161 and 37.03, respectively. The result for predicting acidity was not satisfactory, probably due to the low acid content in the samples. Yu *et al.* [71] applied reflectance spectra in the 400–1075 nm wavelength range to predict sugar content and valid acidity in mango fruit. Eighteen PCs were extracted by PLS and employed as inputs of the GA-BPNN to predict sugar content and 17 PCs for valid acidity. The PLS-GA-BP models yielded good predictive results, with *r* of 0.85 and 0.84 and *SEP* of 0.61 and 0.11, respectively, better than those obtained by the PLS-BPNN models. 

Saranwong *et al.* [118] found DM and starch contents increased significantly during ripening, while no obvious differences in individual sugars and fruit density were observed. Interactance spectra of unripe mangos in the 700–1100 nm wavelength range were obtained. For DM, the optimal predictive result was achieved by the MLR model built with 2nd derivative spectra after MSC-treated. Wavelengths of 914, 882, 826 and 954 nm were used for calibration. The *r* was 0.96 and *SEP* was 0.41%. For starch content, a PLS model based on 2nd derivative spectra in the 850–1000 nm range yielded the best result, with *r* of 0.93 and *SEP* of 1.71%. Further, DM and starch contents in unripe mangos could be used to predict SSC in ripe ones. The best prediction result was achieved by MLR, with *r* of 0.92 and *SEP* of 0.55%. The calibration equation was: SSC = 14.755 + 0.812 DM + 0.677 starch.

Jha *et al.* [119] proposed a maturity index (*I*_m_) based on seven mango cultivars:

$$I_{m}=\eta \frac{\text{SSC}\times \text{DM}}{\text{TA}}\;$$

where η represents a constant specific for each cultivar. *I*_m_ was field tested with less than 10% variation. *I*_m_ could be predicted by using a PLS model based on spectra in the 1600–1800 nm wavelength range, with *r_p_* of 0.68 and *SEP* of 0.34.

### 3.10. Other Fruits

Roger and Bellon-Maurel [9] predicted sugar content in cherry fruit using spectra in the 800–1100 nm wavelength range. A The PLS model based on spectra processed with moving average smoothing yielded a result with *r* of 0.95 and *RMSEP* of 3.43°Brix. A better result could be obtained employing CWs selected by GA, with *r* of 0.98 and *RMSEP* of 0.91°Brix. This result was in the same level with that reported by Lu [120], who applied reflectance spectra in the 800–1700 nm wavelength range. The PLS models they built yielded an *r_p_* and *SEP* of 0.95 and 0.71°Brix for ‘Hedelfinger’, and 0.89° and 0.65°Brix for ‘Sam’. They also predicted their firmness, with *r_p_* of 0.80 and 0.65 and *SEP* of 0.79 N and 0.44 N respectively.

Paz *et al.* [12] applied Vis/NIR reflectance spectra to predict SSC and firmness in plum. SSC could be predicted using a modified PLS model based on spectra in the 515–1400 nm range, with *r* of 0.88 and *SECV* of 0.83°Brix. Firmness could only be predicted using the PLS model built with 515–1650 nm spectra, with *r* of 0.72 and *SECV* of 2.54 N.

Zhang *et al.* [121] predicted soluble tannin content in persimmon using diffuse reflectance spectra in the 570–1848 nm wavelength range. Different pretreatment methods and calibration methods were compared and the best performance was achieved by the modified PLS model based on 1st derivative spectra processed with de-trending, with *r_p_* and *RMSEP* of 0.82 and 0.18 respectively. More research should be done to enhance the prediction accuracy.

## 4. Conclusions and Future Research

For their prominent advantages such as simultaneous, precise and rapid analyses compared to traditional methods, spectroscopy, multispectral imaging and hyperspectral imaging have been widely utilized for the measurement of internal and external quality attributes of fruits. One important evaluation criterion for the successful implement of a spectroscopy technique is the accuracy and robustness of the calibration model. According to the overviews above, further studies should be focused on these aspects rather than doing some superficial research or repeating previous studies:

(1) The optimal spectral acquisition condition, as well as preprocessing and calibration method for each kind of fruit needs to be figured out.
(2) A large database is crucial, for stable and accurate models should yield satisfactory performance even when applied to fruit from different origins, seasons and climate conditions.
(3) The model transference between different types of spectrometers hasn’t attracted enough attention yet.
(4) Most of the papers published focused on several major attributes including SSC, acidity and firmness, other important nutrient compositions such as vitamin content, mineral substance and pigments haven’t attract enough attention.
(5) The feasibility of using Vis/NIR spectroscopy to predict some quality attributes has been verified, but the prediction for some other attributes remains uncertain or is definitely less accurate.

Hyperspectral imaging, combining the advantages of imaging technology and spectroscopy technology, could provide abundant information related to fruit quality and thus offers exciting new possibilities. Although hyperspectral imaging with chemometrics frees researchers from laborious measurements and burdensome computations during food quality assessment, hyperspectral imaging has not been applied for online detection, which is restricted by its massive data volume, different prediction results of spectra mode, external characteristics of samples and expensive equipment [4,5,77,78]. Qin and others [122] established a small-scale hyperspectral reflectance imaging for real-time detection of grape canker, but the system only provides a small number of observations from the whole fruit. Multispectral imaging based on selected critical wavelengths derived from hyperspectral imaging has received great attention. Due to their relative little spectral data, low instrument cost and high analytical speed, multispectral imaging systems could be widely used in online detection and practical applications for fruits [78,98,103]. Huang and others [78] selected three effective wavelengths 750, 820 and 960 nm to realize multispectral imaging tests and obtained good prediction results. However, in consideration of the limitations of multispectral imaging, few selected and discrete wavelengths, multispectral imaging has relatively worse performances on the detection of fruit characteristics, such as firmness [98]. However, with the improvement of computer resources, broad prospects are expected. With the help of NIR microscopes and Raman spectroscopy, observation and detection could be achieved at the histological and cellular level [123,124,125]. A great many of technologies and problems require urgent study and solutions.

Although there is a load of existing problems, the emergence of new technologies and new devices is bringing huge potential to this field. The improved acquisition speed and simplified operation of newly developed spectrographs, multispectral imaging systems and hyperspectral imaging systems combined with the implementation of effective chemometric methods, such as PLS and LS-SVM, have finally make the idea of online detection possible. However, although some efforts have been done to build online detection systems [76,84], real mature and feasible systems are not available in market due to various problems including expensive price, unstable models and complex operation. There is plenty of research left for us to do.
